# Pupal Exuviae of *Culex Pipiens* L. (Diptera: Culicidae) Can be Utilised as a Non-Invasive Method of Biotype Differentiation

**DOI:** 10.1186/s12575-024-00246-1

**Published:** 2024-06-18

**Authors:** Laura Jones, Christopher Sanders, Marion England, Mary Cameron, Simon Carpenter

**Affiliations:** 1 The Pirbright Institute, Ash Road, Woking, Surrey GU24 0NF England; 2 London School of Hygiene and Tropical Medicine, Keppel Street, London, WC1E 7HT England; 3 School of the Biological Sciences, University of Cambridge, 17 Mill Lane, Cambridge, CB2 1RX England

**Keywords:** Arbovirus, Pupae, Biotype, Molestus, DNA Extraction, Direct PCR

## Abstract

**Background:**

*Culex pipiens* L. is a principal vector of zoonotic arboviruses in Europe, acting in both an amplification role in enzootic transmission between avian hosts and as a bridge vector between avian hosts and mammals. The species consists of two forms which are indistinguishable using morphological methods but possess varying ecological and physiological traits that influence their vector capacity. In this study we validate methods that can be used to extract trace DNA from single pupal exuviae of *Cx. pipiens* for use in molecular speciation of samples. These DNA extraction methods are compared using measurement of the total yield and successful identification using a real-time polymerase chain reaction (PCR) assay.

**Results:**

Genomic DNA was initially extracted from colony-derived individuals using an ethanol precipitation method, two commercially available DNA extraction kits: DNeasy® Blood & Tissue Kit (Qiagen, UK) and Wizard® SV Genomic DNA Purification System (Promega, UK) and a direct real-time PCR method. Time elapsed between eclosion and processing of pupae significantly influenced *Cx. pipiens* form identification as nucleic acid concentration and PCR amplification success decreased with increased time elapsed. Real-time PCR amplification success, however, was not shown to vary significantly between the three extraction methods, with all methods successfully identifying all samples, but the direct real-time PCR method achieved a lesser amplification success rate of 70% (*n* = 20 for each treatment). More variable results were produced when field-derived exuviae were used, with no significant difference in real-time PCR amplification success found across the four methods and a lower overall rate of successful identification of 55–80%.

**Conclusions:**

This study shows that both colony and field derived *Cx. pipiens* pupal exuviae can be a useful non-invasive source of trace DNA permitting accurate biotype differentiation for at least twenty-four hours post-eclosion. The significance and utility of this technique in ecological and behavioural studies of *Cx. pipiens* is discussed and recommendations made for use according to experimental scenario.

**Supplementary Information:**

The online version contains supplementary material available at 10.1186/s12575-024-00246-1.

## Background

Vector-borne diseases have a major global impact on the health of human and animal populations. More than 17% of all infectious diseases in humans are transmitted by insects and ticks [1], whilst in livestock, one-quarter of pathogens causing a notifiable disease, as defined by the World Organisation for Animal Health (WOAH, founded as OIE), are vector-borne [2]. *Culex pipiens* Linnaeus 1758 is a vector of pathogens of global importance, including arborviruses (e.g. West Nile and Usutu viruses), protozoa (e.g. avian malaria) and dirofilarial worms [3–5]. The *Cx. pipiens* species comprises two biotypes that cannot be separated by morphology: *Cx. pipiens* f. *pipiens* (L.) and *Cx. pipiens* f. *molestus* Forskål 1775. Traditionally, physiological and behavioural traits including stenogamy, autogeny and host preference have been used to differentiate the biotypes [6]. This variation was hypothesised to result from adaptation to different habitats, with the *Cx. pipiens* f. *molestus* biotype being primarily found in underground environments and *Cx. pipiens* f. *pipiens* occurring above-ground [7, 8]. More recent analysis of *Cx. pipiens* populations in Europe, however, indicates a far more complex scenario, with inter-breeding of above- and below-ground sympatric populations and the occurrence of hybrids that exhibit variation in physiological and behavioural phenotype [9–15].

Species identification methods utilising target organism DNA or RNA are now a fundamental tool for study of vector populations and the pathogens they transmit [16]. The lack of clarity in classification of *Cx. pipiens* biotypes has led to application of molecular methods being applied more frequently in differentiating these forms, predominantly through amplification of the CQ11 microsatellite locus [9, 17]. Molecular methods of taxonomic identification are typically lethal for the individual sampled, or at a minimum involve the removal of body parts with subsequent impact on behaviour and survival [18]. In recent years, however, non-lethal sampling methods for DNA extraction have gained popularity across a wide range of subject areas including conservation, behaviour and population genetics [19]. Previously, a single tibia has been shown to be a sufficient non-lethal source of DNA in several insect species including bumble bees [20] and mosquitoes of the *Anopheles* genus [21]. Haemolymph, wing edges and tips have also been shown to provide a sufficient source of DNA for identification in other insect species [18, 22]. These methods are fundamentally inappropriate, however, for behavioural studies that rely on minimal disturbance such as studies of leg tapping within the *Cx. pipiens* group, where females are observed to perform rejection kicks to prevent mating with incompatible males [23, 24].

Collection and identification of insect pupal exuviae has been an important tool in many ecological studies of insects aiming to estimate population densities [25], species distribution [26, 27] and adult emergence periods [28]. Successful extraction of DNA has been demonstrated for a variety of invertebrates including from the exuviae of butterflies [29], honey bees [30], mosquitoes [31], dragonflies [32], scarabs [33] and from the moults of tarantulas [34]. The exoskeleton of the pupal exuviae is comprised of extracellular chitin and does not itself contain any nucleic acids [35], but trace quantities of epithelial cells, hairs and muscle tissues from the adult remain attached to the inner surface of the cuticle [35]. Trace DNA from chironomid pupal exuviae has been shown to be sufficient for PCR amplification and sequencing of the COI barcoding gene in 46% of samples tested [36]. A further study isolated genomic DNA from an average of 61.2% of chironomid samples tested across five different extraction methods, with varying success depending on the method used [37]. Pupal exuviae from *Aedes* and *Culex* mosquitoes were used successfully to obtain DNA for molecular speciation targeting the ITS2 and ITS1 regions, respectively, although success was limited to within the first 24 h post-eclosion and no success was seen when tested in field collected samples [31]. A subsequent study was unable to obtain enough DNA from pupal exuviae of *Aedes aegypti* (L. 1762) for downstream molecular testing [38].

The present study aims to assess the utility of *Cx. pipiens* pupal exuviae as a non-lethal source of DNA for distinguishing the two *Cx. pipiens* biotypes from mixed populations under both laboratory and field conditions. The effect of processing method on DNA yield and PCR amplification success from exuviae samples was investigated, including an assessment of handling time and cost. Processing time post-eclosion on DNA yield and PCR amplification success was also quantified using a mixed biotype colony line. The validated protocol was subsequently applied to field collected exuviae samples and DNA yield and PCR amplification success of samples processed individually and in pools of five exuviae compared. The effect of body size as well as environmental factors, such as light intensity and water temperature, on DNA yield were also investigated. DNA extraction from pupal exuviae was then used to establish single biotype colonies from a mixed population and to estimate how long these colonies remain pure in culture, as a tool to facilitate behavioural studies between the two biotypes.

## Methods

### Mosquito Rearing

Experiments utilised a mixed colony of *Cx. pipiens* biotypes originating from an allotment area in Brookwood, Surrey, UK established in 2011 and identified as the ‘Brookwood’ line [11]. Five to seven days old adults from this colony line were offered defibrinated horse blood (TCS Biosciences, UK) overnight using a Hemotek™ membrane feeding system (Hemotek™ Ltd., UK). Five days after blood feeding, egg rafts were collected from oviposition cups and left to hatch in approximately 500 ml of fresh tap water. Larvae were reared at a density of 200 larvae/L water and maintained on a diet of 1 mg of guinea pig pellets/larva (Pets at Home, UK) on alternate days. Larvae were reared in an environmentally controlled incubator maintained at a temperature of 25^o^C ± 1^o^C and relative humidity of 50% ± 1% and were exposed to a lighting regime of 16:8 h light: dark. Conditions in the incubator were monitored using a HOBO™ U12-012 temperature/relative humidity/light data logger (Measurement Systems Limited, UK). Daylight period light intensity was approximately 3500 lx whilst light intensity during the night-time period was 3.5 lx. Dusk and dawn were simulated by either an increase or decrease in light intensity, respectively, each over a one-hour period.

### Pupal Exuviae Sample Collection and Preparation

Following the onset of pupation, pupae were collected daily and placed into separate 2 ml tubes containing 1 ml of water and monitored for eclosion. Pupal exuviae and eclosing adults were collected and used for two different experiments. Firstly, to compare the efficacy between DNA extraction methods, pupal exuviae and emerged adult mosquitoes were placed in 70% ethanol immediately following eclosion and stored individually at room temperature prior to DNA extraction. Secondly, to assess the effect of time elapsed between eclosion and preservation on DNA extraction efficiency, adult mosquitoes and their corresponding pupal exuviae were placed individually into 70% ethanol at defined time points post-eclosion (0, 1, 6, 12, 18 and 24 h). Pupal exuviae and adult mosquitoes were then removed from ethanol and left to air dry for approximately 10 min prior to DNA extraction. Comparisons of DNA quantity between different processing times post-eclosion were assessed using Kruskal-Wallis with Bonferroni correction for multiple comparisons. For all statistical tests, the statistical significance level was set at *P* < 0.05 and analyses were computed in R studio version 1.2 [39].

### Use of Pupal Exuviae as a Non-Invasive Method of Sampling Field Populations

Two periods of pupal exuviae collection were undertaken in the field, each spanning two weeks. The first collections were performed in August 2020 and used for comparison between individual and pooled processing approaches. The second collection period was between June to July 2021 and used to validate processing methods in field collected samples. The oviposition site in the field was created using a 20 L polypropylene black bucket of 28.3 cm x 47.8 cm x 33.0 cm (HxWxD), filled with 10 L of tap water and seeded with 5 g crushed guinea pig pellets (Pets at Home, UK) to attract gravid female *Cx. pipiens*. The bucket was placed in a residential garden in Guildford, Surrey (51°240’N; -0°578’W). Pupal exuviae floating on the surface were collected and placed into 70% ethanol each morning over a period of two weeks and transferred to the laboratory for processing. Samples were either processed individually or in pools containing five exuviae using the ethanol precipitation method described below. Water temperature and light intensity in the bucket were measured using a HOBO™ temperature/light weatherproof pendant data logger (Measurement Systems Limited, UK). Mann-Whitney U tests were used to assess the effect of pooling or year of collection on nucleic acid concentrations from field collected pupal exuviae.

### DNA Extraction Method Comparison

Three extraction methods and a direct real-time PCR processing method were chosen to compare DNA yield and subsequent PCR amplification success: an ethanol precipitation method and two commercially available kits, DNeasy® Blood & Tissue Kit (Qiagen, UK) and Wizard® SV Genomic DNA Purification System (Promega, UK), hereafter referred to as DNeasy® and Wizard® respectively. For each of the extraction methods, total genomic DNA was isolated from 20 individual pupal exuviae alongside a distilled water negative control, with ten males and ten females processed for each method. DNA extracted using the same method from the corresponding adult heads were additionally included as positive controls.

#### Ethanol Precipitation

DNA was extracted as follows: samples were placed into 200 µl of digestion solution comprised of 100 mM UltraPure™ 1 M Tris-HCL (pH 8.0) (Invitrogen™ by Thermo Fisher Scientific, UK), 200 mM NaCl (Invitrogen™, UK), 0.2% (w/v) SDS (Merck Life Science UK Limited, UK), 5 mM UltraPure™ 0.5 M EDTA (pH8.0) (Invitrogen™, UK), 200 µg/ml proteinase K (Qiagen, UK) made up to a total volume of 200 µl per sample using UltraPure™ water (Invitrogen™, UK). Samples were incubated overnight at 37^o^C before proceeding with DNA extraction. Immediately following incubation, 500 µl of ice-cold 100% ethanol, 20 µl 3 M NaOAc pH 5.5 (Invitrogen™, UK) and 2 µl of GlycoBlue™ coprecipitant (15 mg/ml) (Invitrogen™, UK) were added to each sample and incubated at -20^o^C for 1 h. DNA was pelleted by centrifugation at 14,000 rpm at 4^o^C for 30 min and supernatant was removed. Pellets were then washed in 400 µl 70% ethanol, re-pelleted by centrifugation under the same conditions for 15 min and supernatant was removed. Pellets were air-dried for 20 min to allow for the evaporation of excess ethanol and resuspended in either 15–200 µl nuclease free water for pupal exuviae and adult samples respectively.

#### Commercially Available Kits

DNA was extracted according to the manufacturer’s instructions with the following modifications to enhance DNA yield [40, 41]. Mosquito exuviae were subjected to a homogenisation stage within the digestion solution detailed in each of the kit’s instructions using 3 mm stainless steel homogenisation beads (Qiagen, UK) for 1 min at 30 Hz using a Qiagen tissue lyser (Qiagen, UK). Samples were then incubated overnight at 56^o^C prior to DNA extraction. To maximise DNA yield from exuviae samples, 30 µl of elution buffer was incubated on the spin column membrane at room temperature for 5 min prior to elution. For DNA extraction from mosquito heads, elution was performed twice in 30 µl for a total elution volume of 60 µl.

#### Direct Real-Time PCR

Samples were prepared according to Thongjued et al. [42] with minor adjustments to sample preparation. Briefly, pupal exuviae were removed from ethanol and left to air-dry for approximately 10 min before being placed in 20 µl PBS (pH 7.4) (Gibco™ by Thermo Fisher Scientific, UK). Samples were vortexed before incubating at 98^o^C for 4 min and 2 µl of supernatant was added directly to the PCR mix.

In all samples, prior to PCR processing, nucleic acid concentrations were measured for all extraction methods using the Qubit® dsDNA HS assay kit (Invitrogen™, UK) and read by a Qubit® 3.0 fluorometer (Invitrogen™, UK). For all samples, 2 µl of DNA template were used in 198 µl of the dsDNA HS assay. Variation in DNA yield between processing methods for both colony and field collected exuviae were assessed using a Kruskal-Wallis with Bonferroni correction for multiple comparisons.

### Differentiation of *Cx. Pipiens* Biotypes

Mosquitoes were simultaneously assigned to species and biotype level using a real-time PCR assay originally designed by Rudolf et al. [9] with minor adaptations to primer concentrations. Reactions were performed in 10 µl reaction volume consisting of 5 µl TaqMan™ multiplex master mix (2x) (Applied Biosystems™ by Thermo Fisher Scientific, UK), 0.3 µM *CxPip*F, 0.4 µM *CxPip*R, 0.2 µM *CxPip*P, 0.2 µM *CxPipPip*P, 0.2 µM *CxPipMol*P, 0.15 µM *CxTorr*F, 0.15 µM *CxTorr*R R, 0.1 µM *CxTorr*P, 0.16 µl BSA (20 mg/ml) (Merck Life Science UK Limited, UK), 1.14 µl UltraPure™ water (Invitrogen™, UK) and 2 µl DNA extract. Sequences for the primers and probes are shown in Table 1. The thermal profile started with an initial activation step of 95^o^C for 20 s, followed by 40 cycles of 95^o^C for 3 s and 60^o^C for 1 min using a QuantStudio™ 7 Flex Real-Time PCR machine (Applied Biosystems™, UK). All samples were run alongside positive controls consisting of pure f. *pipiens*, f. *molestus*, hybrid and *Cx. torrentium* DNA as well as negative controls including extraction and PCR negative controls. Real-time PCR amplification success was defined by the number of samples for which the quantification cycle (C_q_) value was below the assay cutoff of 39. Any samples not producing a C_q_ value were assigned a value of 40 for analysis. C_q_ values obtained from each of the probes for each sample were averaged for use in analysis. Variation in PCR amplification success between processing time post-eclosion or processing method was assessed with pairwise chi-squared tests for independence with Bonferroni adjustment for multiple comparisons.

**Table 1:** Primer and probe sequences for the simultaneous differentiation of *Cx. pipiens* and *Cx. torrentium* species through amplification of the ACE-2 gene as well as the *Cx. pipiens* biotypes by the CQ11 microsatellite locus. Letters in the primer/probe names identify whether they are forward (F) primers, reverse (R) primers or probes (P)

Primer/probe name	Primer/probe sequence
*CxPip*F	5’- GCGGCCAAATATTGAGACTT-3’
*CxPip*R	5’-CGTCCTCAAACATCCAGACA-3’
*CxTorr*F	5’-GACACAGGACGACAGAAA-3’
*CxTorr*R	5’-GCCTACGCAACTACTAAA-3’
*CxPip*P	5’-VIC- GGAACATGTTGAGCTTCGG-QSY-3’
*CxPipPip*P	5’-ABY-GCTTCGGTGAAGGTTTGTGT-QSY-3’
*CxPipMol*P	5’-JUN-TGAACCCTCCAGTAAGGTATCAACTAC-QSY-3’
*CxTorr*P	5’-FAM-CGATGATGCCTGTGCTACCA-QSY-3’

### DNA Extraction Cost and Handling Time

The cost of the extraction method for each sample was estimated based on the retail price of the chemicals or kits used in the UK. The handling time for each sample was calculated as the time required to complete all processing, starting from dehydration of the pupal exuviae, to obtaining a DNA extract ready for PCR. This was replicated three times with each round containing 10 individuals and averaged.

### Wing Length Measurements

**Fig. 1:**
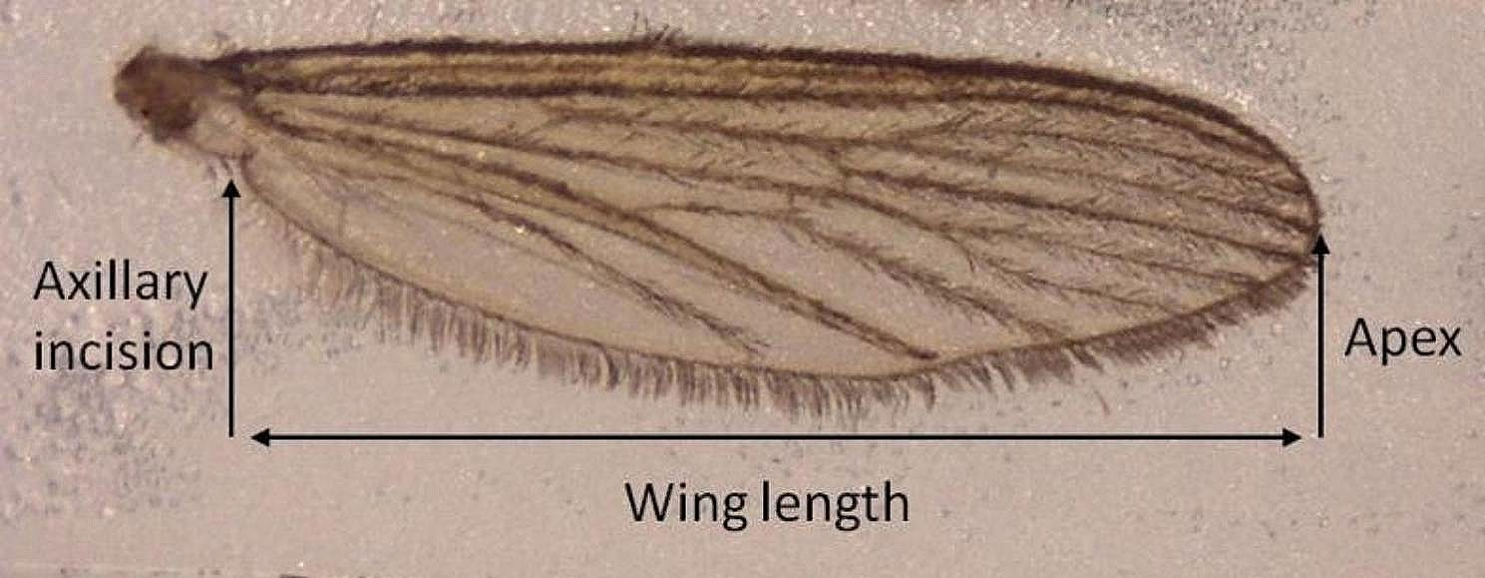
Wing length measurements of *Cx. pipiens* mosquitoes used as a proxy for body size. Measurements are taken from the axillary incision to the apical margin, excluding the fringe of the wing

Wing length was measured as a proxy for body size to control for specimen size variation for the method comparison and time trial experiments [43]. Wings from sampled individuals were transferred to a piece of paper towel dampened with 70% ethanol, flattened, and left to air dry for approximately 5 min. Wings were then transferred to a strip of Scotch Magic Tape™ with both wings from one adult placed together and placed on a glass microscope slide for imaging. Wing images were visualised using a Leica EZ4HD microscope (Leica Microsystems, Germany) alongside a stage micrometer for scale where one division is equal to 0.01 mm. Images were subsequently processed for size measurement using ImageJ [44]. Wing length was measured from the axillary incision to the apical margin, excluding the fringe (Fig. 1) [45]. Measurements were taken from both wings of the same adult and averaged for use in analysis. Correlations between concentration, wing measurement and C_q_ values were assessed using Spearman’s correlation.

### Creation of Single Biotype Colony Lines

Approximately five days after blood-feeding, egg rafts were collected from the Brookwood colony line (F97) and each egg raft separated into individual 25 ml pots in approximately 15 ml of dechlorinated tap water for hatching. Larvae from each egg raft were reared separately under the same conditions described above. Following the onset of pupation, pupae were collected daily into individual 2.0 ml tubes and monitored for eclosion. Pupal exuviae were collected into 70% ethanol every twelve hours and stored prior to processing. Exuviae from emerging adults were processed for DNA extraction by ethanol precipitation and biotype identified daily by real-time PCR as described above. PCR results were used to allocate adult mosquitoes to separate colony cages (Bugdorm, Watkins and Doncaster, UK) according to the biotype of their corresponding exuviae with approximately 80–100 individuals per biotype used to create single biotype colonies. The colony lines were subsequently maintained according to Manley et al. [11]. After 10 generations, DNA was extracted from 40 adults from each line using the Wizard® kit and biotype identified by real-time PCR as described to confirm the lines remained homologous or heterozygous as determined by the CQ11 microsatellite marker. Heads of adult mosquitoes were used to obtain genomic DNA using the Wizard® SV Genomic DNA Purification System (Promega, UK) and biotype characterised, as described above.

## Results

### Comparison of Processing Methods for DNA Extraction and Biotype Identification in Colony Populations

**Fig. 2:**
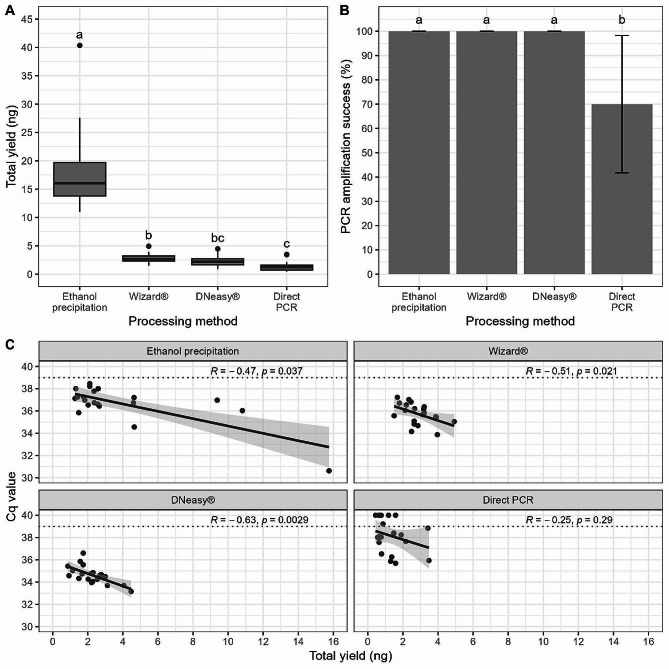
Comparison of three DNA extraction methods and a direct PCR assay from individual *Cx. pipiens* pupal exuviae. **A**) Comparison of total DNA quantity (ng) obtained from individual *Cx. pipiens* pupal exuviae. Horizontal black lines indicate median, 25th and 75th percentiles, whiskers extend to the largest and smallest values within 1.5 times the interquartile range from the 25th and 75th percentiles, closed dots indicate outliers. **B**) Comparison of PCR amplification success (%) for DNA extracts. Error bars indicate mean ± SD. The letters above the bars and boxplots indicate which groups differ significantly (*P* < 0.05) from one another. Specifically, groups which share a common letter are not significantly (*P* < 0.05) different from one another. C) Comparison of total yield (ng) compared with average C_q_ values following PCR amplification of DNA extracts. Grey shaded area indicates the 95% confidence interval

Genomic DNA was successfully extracted from a total of 80 samples (*n* = 20 for each method). The quantity of DNA extracted was shown to vary significantly between the four processing methods (KW χ^2^ = 57.144, df = 3, *P* < 0.001; Fig. 2A). The greatest quantity of total DNA yield was achieved using the ethanol precipitation method (x̅ = 17.780 ng; sd = 6.874 ng) which was significantly greater (*P* < 0.001) than yields from both the DNeasy® (x̅ = 2.193 ng; sd = 1.149 ng) and the Wizard® (x̅ = 3.015 ng; sd = 0.999 ng). The two commercial kits did not differ significantly in their yield (*P* = 0.145). The direct PCR method generated the lowest DNA yield (x̅ = 1.35 ng; sd = 0.864 ng) which was significantly less than quantities obtained from the Wizard® (*P* = 0.005) and highly significantly less than ethanol precipitation (*P* < 0.001) methods but did not differ from the DNeasy® kit (*P* = 0.178).

Despite the differences in total DNA yields obtained through the four methods, PCR amplification success was not shown to vary significantly between the three extraction methods used (Fisher’s exact *P* = 1.00) with all showing 100% amplification success. PCR amplification success for the direct PCR method was significantly reduced, however, when compared with the other extraction methods tested (*P* = 0.007) with an amplification success rate of 70% (Fig. 2B). A general trend was observed in decreasing C_q_ values with increasing DNA yields (Fig. 2C). This correlation was significant for the ethanol precipitation (*R*= -0.47, *P* = 0.037), Wizard® (*R*=-0.51, *P* = 0.021) and DNeasy® (*R*= -0.75, *P* = 0.018) methods. The correlation between total DNA yield and C_q_ value was not significant for the direct PCR method (*R*= -0.25, *P* = 0.29).

### DNA Extraction Cost and Handling Time

The direct PCR method was the most inexpensive method with almost no associated extraction costs, followed by the ethanol precipitation which involved purchase of individual reagents (Table 2). The DNeasy® and Wizard® kits had a higher handling cost per sample with the Wizard® kit having an almost five-fold increase in price per sample compared with the ethanol precipitation. Moreover, the cost of the DNeasy® kit was substantially higher; almost double the Wizard® kit per sample (Table 2). Increased processing costs were associated with decreased C_q_ values (*R* = -0.76, *P* < 0.01) and therefore higher PCR amplification success. Despite this, when considering methods which involved extraction of samples prior to PCR, these higher processing costs did not enhance DNA yield, rather, a significant negative correlation between increased processing costs and DNA yield was detected (*R* = -0.8, *P* < 0.01). When considering the methods that required prior extraction of samples before PCR, decreased cost was correlated significantly with increased processing time (*R* = -0.95, *P* < 0.01).

The direct PCR was the quickest processing method, with no requirement for prior extraction of samples therefore eliminating the overnight incubation step. Of the three extraction methods tested, the ethanol precipitation possessed the longest processing time with the DNeasy® and Wizard® kits taking considerably less time, approximately 2 h less each (Table 2). A significant positive correlation was detected between handling time and DNA yield, with the longer the time spent preparing the samples, the higher the DNA yield prior to PCR amplification (*R* = 0.8, *P* < 0.01). Processing time was also negatively correlated with C_q_ values (*R* = -0.12, *P* = 0.293), therefore increasing PCR amplification success, however, this interaction was not shown to be significant.

**Table 2:** Comparison of cost per sample and processing time of three different extraction methods and a direct PCR for DNA extraction from *Cx. pipiens* pupal exuviae

Method	Cost per sample	Processing time (Hours)	
		Mean	Std dev
Qiagen DNeasy® Blood & Tissue kit	£4.36	18.5	0.025
Promega Wizard® SV Genomic DNA Purification System	£2.32	18.7	0.040
Ethanol precipitation	£0.48	20.6	0.087
Direct PCR	0.06p	0.33	0.026

Prices in 2024

### Investigating the Influence of Processing Time Post-Eclosion on DNA Yield and PCR Amplification Success from *Cx. Pipiens* Pupal Exuviae from Colony Populations

**Fig. 3:**
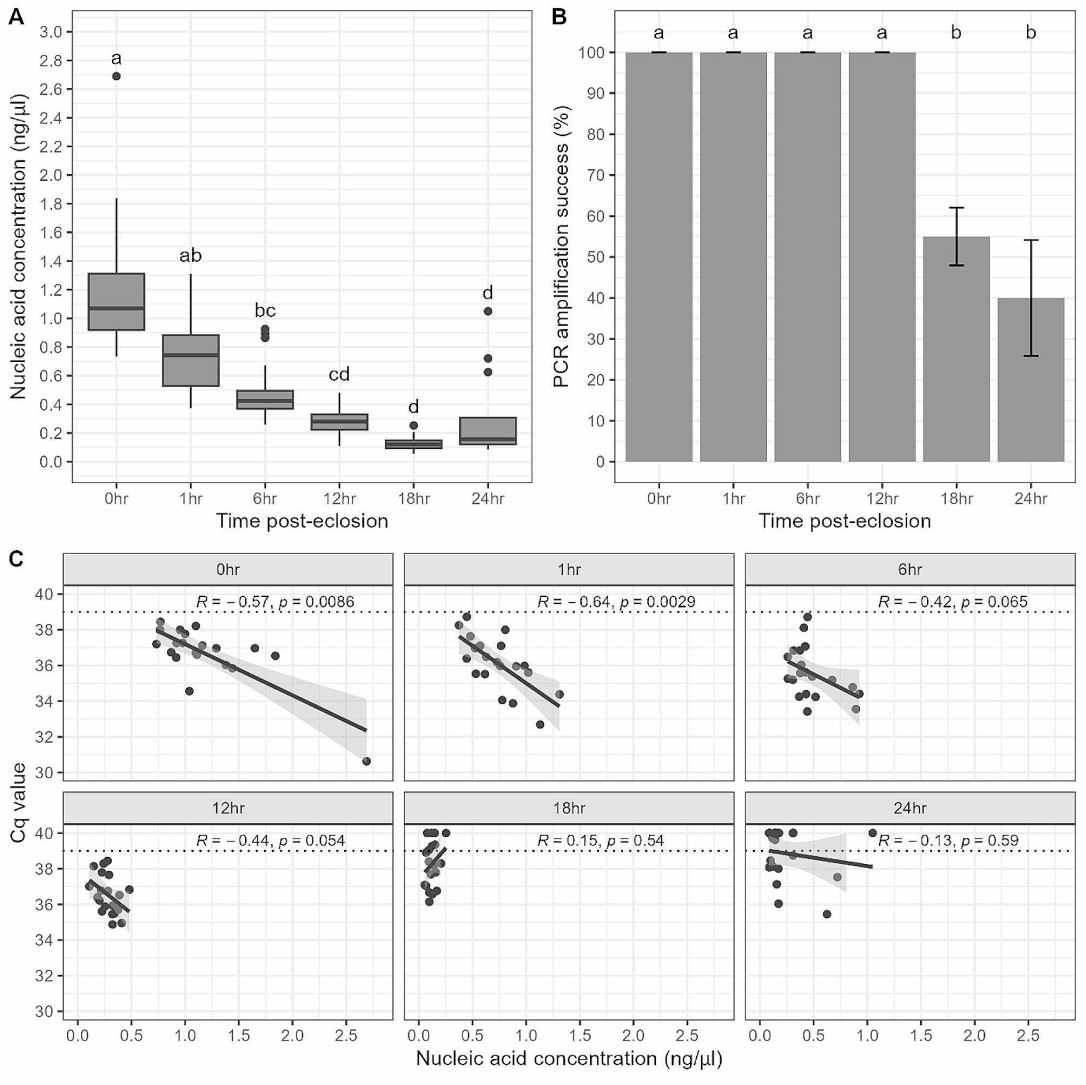
Comparison of DNA yield and amplification success of individual *Cx. pipiens* pupal exuviae at varying time points post-eclosion. **A**) Comparison of DNA quantity (ng/µl) from all DNA extractions using the ethanol precipitation method. Horizontal black lines indicate median, 25th and 75th percentiles, whiskers extend to the largest and smallest values within 1.5 times the interquartile range from the 25th and 75th percentiles, closed dots indicate outliers. **B**) Comparison of PCR amplification success (%) of DNA extracts. Error bars indicate mean ± SD. C) Comparison of nucleic acid concentrations (ng/µl) with mean C_q_ values for DNA extracts. Grey shaded area indicates the 95% confidence interval. Different letters above the boxplots and bars indicate which groups differ significantly (*P* < 0.05) from one another

Genomic DNA yield was found to vary significantly between the different time points post-eclosion (KW χ^2^ = 92.125, df = 5, *P* < 0.01; Fig. 3A). The greatest DNA yield resulted from samples processed immediately after eclosion (x̅ = 1.190 ng/µl; sd = 0.458 ng/µl) which were found to be significantly higher than all other groups (*P* < 0.05) with the exception of the 1-hour time point (*P* = 1.00). Generally, there was a negative correlation between time of processing post-eclosion and DNA yield with mean nucleic acid concentrations decreasing as time passed.

The time that elapsed between eclosion and preservation was shown to significantly influence PCR amplification success (Fisher’s exact = *P* < 0.001). No significant difference was observed between amplification success in samples preserved up to and including 12 h post-eclosion (*P* = 1.00) with all samples showing amplification. In contrast, samples preserved at 18–24 h post-eclosion demonstrated significantly lower PCR amplification success (55% and 40%) compared with samples preserved within 12 h of eclosion (*P* = 0.0184 and *P* < 0.001 for 18 and 24 h respectively). Similarly, a decrease in C_q_ value as nucleic acid concentration increased (Fig. 3C) was only significant for the first two time points (*R* = -0.57, *P* = 0.0086 and *R*= -0.64, *P* = 0.0029 for 0 h and 1 h respectively).

### Effect of Body Size on Nucleic Acid Concentration

Wing length as a proxy of body size, was shown to be positively correlated with nucleic acid concentrations recovered from exuviae (t = 2.62, df = 111, *P* = 0.01) and this interaction was shown to be significant in each time group except for the 18 and 24-hour time points (Figure S1B). There was no significant variation in wing length between groups of mosquitoes included in each treatment (F = 0.172, df = 5, *P =* 0.972), in different *Cx. pipiens* forms (F = 1.458, df = 2, *P* = 0.237; Figure S1A) or in the left and right wing length recorded for each individual (t = 13.737, df = 224, *P* = 0.956).

### Comparison of Processing Methods for DNA Extraction and Biotype Identification from Field Collected Pupal Exuviae

Genomic DNA yield varied significantly between individually processed and pooled field exuviae samples (W = 16.6, *P* < 0.001; Figure S2A) when tested using the ethanol precipitation method. The greatest DNA quantities resulted from the pooled samples (x̅ = 1.000 ng/µl; sd = 0.181 ng/µl), yet results showed that DNA yield from individually processed samples was still sufficient for detectable results, despite yielding lower concentrations (x̅ = 0.243 ng/µl; sd = 0.141 ng/µl). Amplification success was also affected significantly by pooling as 100% of pooled samples successfully amplified target sequence, significantly higher than the 55% recorded in individually processed samples (Fisher’s exact *P* = 0.0292; Figure S2B). Nucleic acid concentration was shown to be correlated with C_q_ value for both individually processed and pooled samples with C_q_ values shown to decrease as nucleic acid concentrations increased (Figure S2C).

### Comparison of Three DNA Extraction Methods and a Direct Real-Time PCR Assay for Field Collected Pupal Exuviae

**Fig. 4:**
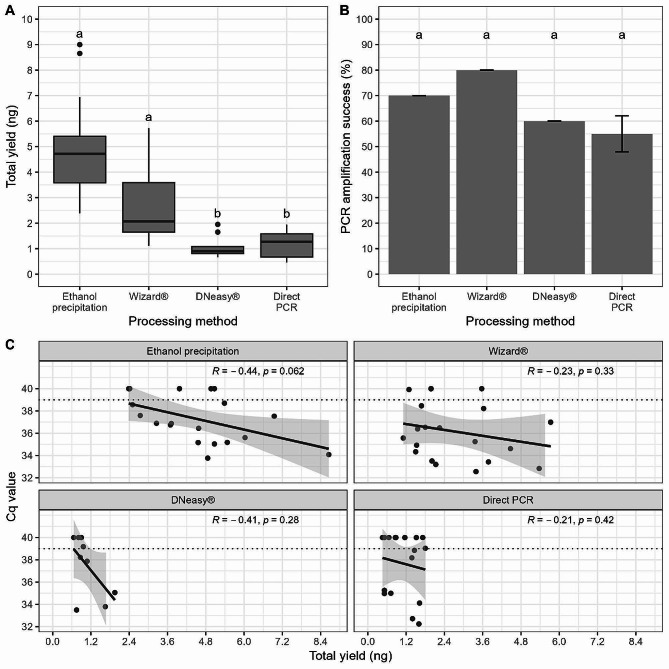
Comparison of the efficiency of three extraction methods for obtaining DNA from individual field collected *Cx. pipiens* pupal exuviae and a direct PCR method that did not include an extraction step. **A**) Comparison of total DNA quantity (ng) from DNA extractions. Horizontal black lines indicate median, 25th and 75th percentiles, whiskers extend to the largest and smallest values within 1.5 times the interquartile range from the 25th and 75th percentiles, closed dots indicate outliers. **B**) Comparison of PCR amplification success (%). Error bars indicate mean ± SD. C) Comparison of total DNA yields (ng) with mean C_q_ values for different processing. Grey shaded area indicates the 95% confidence interval. Different letters above the boxplots and bars indicate which groups differ significantly (*P* < 0.05) from one another

Quantity of DNA obtained from field samples was shown to vary significantly between the four extraction methods (KW χ^2^ = 45.82, df = 3, *P* < 0.001; Fig. 4A). The greatest total DNA quantity was achieved using the ethanol precipitation method (x̅ = 5.17 ng; sd = 1.75 ng) which was statistically greater than both the DNeasy® kit and the direct PCR (*P* < 0.001). The Wizard® kit yielded the second largest DNA quantities which again were significantly greater than both the DNeasy® (*P* = 0.00869) and direct PCR (*P* = 0.0276) methods. There were no statistically significant differences in PCR amplification success between the four processing methods (Fisher’s exact *P* = 0.367) although the Wizard® kit yielded the highest rate of amplification success (80%) (Fig. 4B). A correlation of decreasing C_q_ with increasing DNA yields (Fig. 4C) was detected although this was not statistically significant for any of the processing methods.

**Fig. 5:**
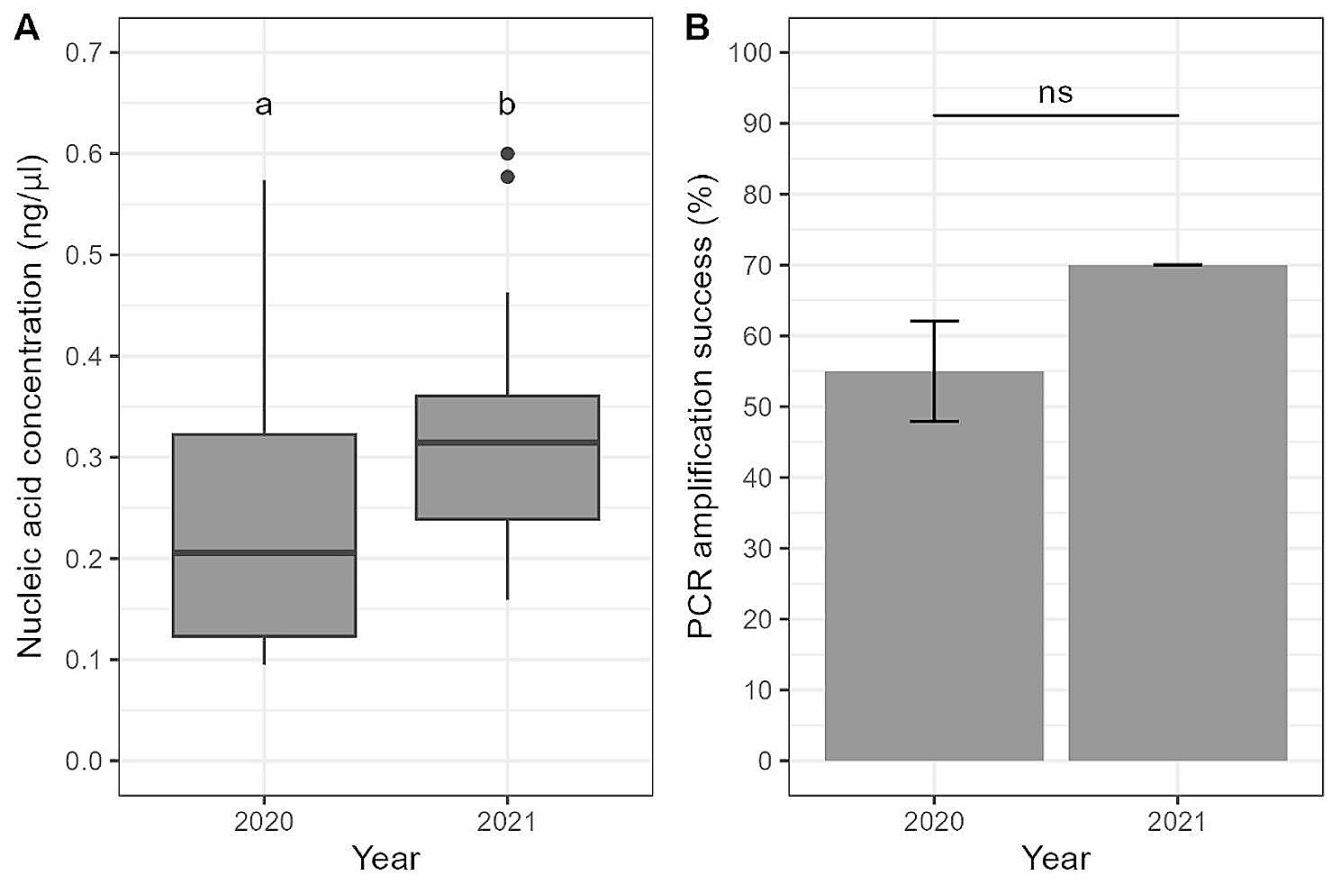
Comparison of nucleic acid concentration and PCR amplification success of individual field collected *Cx. pipiens* pupal exuviae between different sampling years. (**A**) Comparison of nucleic acid concentration (ng/µl) from DNA extracts between different sampling periods using the ethanol precipitation extraction method. Horizontal black lines indicate median, 25th and 75th percentiles, whiskers extend to the largest and smallest values within 1.5 times the interquartile range from the 25th and 75th percentiles, closed dots indicate outliers. (**B**) Comparison of PCR amplification success (%) for two different sampling periods. Error bars indicate mean ± SD. Different letters above the bars indicate which groups differ significantly (*P* < 0.05) from one another

Nucleic acid concentrations obtained using the ethanol precipitation method differed significantly between sampling years (W = 52, *P* = 0.0366), with samples collected in 2021 yielding higher average concentrations compared with those collected in 2020 (x̅ = 0.243 ng/µl; sd = 0.141 ng/µl and x̅ = 0.345 ng/µl; sd = 0.117 ng/µl for 2020 and 2021 sampling period respectively; Fig. 5A). Although amplification success was higher for samples collected in 2021, this difference was not statistically significant (*P* = 0.5402; Fig. 5B).

During 2020, average water temperatures of 21.3 ± 2.71^o^C were significantly higher than those recorded during 2021 (17.7 ± 1.20^o^C; W = 354, *P* < 0.001). Similarly, 2020 recorded significantly higher lux levels (34,444 lx; W = 813, *P* < 0.001) compared with those recorded during collections conducted in 2021 (10,066 lx). All field collected specimens were characterised as *Cx. pipiens* f. *pipiens* according to the CQ11 microsatellite marker.

### Creation of Pure Colony Lines Using DNA Extraction from Pupal Exuviae to Select Live Individuals

Extraction of DNA from pupal exuviae was successfully used to select live individuals to create single biotype colonies from a mixed population, which were reared for more than 10 generations. Mosquitoes tested from the 10th generation demonstrated that the colonies remained stable until this point (Table 3) with the hybrid line consisting of the expected mixture of forms (22.5% f. *pipiens*, 20% f. *molestus* and 57.5% hybrid specimens).

**Table 3:** Biotype results of 40 adult mosquitoes from the 10th generation of each of the three single biotype colony lines created, according to the CQ11 microsatellite marker

	Biotype according to CQ11 PCR		
Colony line	*Cx. pipiens* f. *pipiens*	*Cx. pipiens* f. *molestus*	Hybrid
*Cx. pipiens* f. *pipiens* colony	40	0	0
*Cx. pipiens* f. *molestus* colony	0	40	0
Hybrid colony	9 (22.50%)	8 (20.00%)	23 (57.50%)

## Discussion

In this study, successful DNA extraction and PCR amplification from individual pupal exuviae was demonstrated from colony- and field-reared *Cx. pipiens* populations using three different extraction methods. Extracted DNA was successfully used to accurately identify biotypes within the *Cx. pipiens* species and the method was employed to create single-biotype colony lines from a mixed population which retained their homologous genetic signature according the CQ11 for over 10 generations. The method has wide applicability for future studies of the genetic underpinning of behaviour in *Cx. pipiens* both in the field and laboratory through the provision of non-invasive identification.

Successful DNA extraction and PCR amplification from individual pupal exuviae was demonstrated for up to twenty-four hours post-eclosion, although amplification success was shown to decrease as time elapsed since post-eclosion increased. Time points up to and including twelve hours post-eclosion showed 100% amplification success, but this decreased for the eighteen (55%) and twenty-four-hour (40%) time points highlighting the requirement for relatively rapid processing. This limitation is likely to be due to nucleic acid degeneration and imposes greater limitations in the field where the age of the exuviae processed may be unknown.

Successful DNA extraction and PCR amplification from pupal exuviae of laboratory reared *Culex* and *Aedes* species within the first twenty-four hours post-eclosion has previously been reported [31]. However, no successful amplification was observed after twenty-four hours (and up to nine days) post-eclosion when testing pupal exuviae from colony reared individuals. Contrastingly, a subsequent study attempting to replicate this with *Ae. aegypti* pupal exuviae collected one-hour post-eclosion entirely failed to amplify PCR targets [38]. Neither study quantified the resulting DNA extraction concentrations, therefore it is unknown whether the quantity of DNA used in the PCR was sufficient to meet the lower threshold of the reaction to allow detection. In contrast, the current study utilised a real-time PCR method with a high dynamic range, capable of amplifying small quantities of DNA as low as 2 × 10^− 4^ ng (unpublished data). Real-time PCR methods are likely to have a lower detection threshold compared with the traditional PCR methods used in previous studies, which could in part explain the increased amplification success seen [46]. Further work should determine if DNA yield obtained from methods developed during this study are sufficient for successful amplification in endpoint PCR assays and sequencing enabling use of this method for a broader array of species and scenarios.

Processing methods involving extraction of DNA from samples provided superior concentration of isolated trace DNA from single *Cx. pipiens* pupal exuviae than the direct protocol. Although isolation of DNA by ethanol precipitation had the longest processing time, the DNA quantity was significantly higher than the other processing methods whilst also having the lowest processing cost compared with the other extraction methods. In contrast, DNA extraction success from chironomid pupal exuviae found that commercial kits yielded the best results [37], although a DTAB/CTAB lysis protocol was used rather than ethanol precipitation to compare against commercially available kits. The lower material cost of the ethanol precipitation method may in some scenarios facilitate processing of greater sample sizes. More broadly, selection of the optimal method is dependent on the monetary and time resources available, as well as ease of use and application to a variety of sample types, that may favour the use of commercial kits for some projects.

Despite a reduced amplification success (70% compared with 100%), direct PCR was significantly cheaper and quicker to perform than the other processing methods tested. Although this would not be suitable for highly valuable specimens, direct PCR would be of particular benefit when utilising starting material with higher PCR amplification success rates such as whole larvae [42] as it would facilitate rapid screening of populations at a reduced cost. Significantly higher PCR amplification success (70%) was obtained during the present study compared with similar studies in chironomids [37], attributed to the use of an optimised direct PCR methodology. Incubation of the exuviae in PBS may dilute PCR inhibitors whilst also maintaining the pH of the reaction [47]. Moreover, the heating step within the direct PCR approach aids cell lysis, releasing DNA and denaturing proteins that could degrade DNA or inhibit the PCR [48, 49]. Amplification success rates of 90.5% have been achieved from Diptera specimens when using a high-fidelity DNA polymerase specifically designed for use in direct PCR application with increased tolerance to PCR inhibitors [42]. Thus, further optimisation of this protocol using a high fidelity *Taq* has the potential to be beneficial in increasing PCR amplification success rate.

The present study also demonstrated successful DNA extraction and PCR amplification of field collected pupal exuviae when processed both individually and in pools of five exuviae, with individual processing applicable when comparing multiple processing methods. As expected, processing of exuviae in pools yielded higher nucleic acid concentrations as well as consistent PCR amplification success compared with individually processed exuviae (100% compared with 55%). Successful extraction and amplification from field collected insect exuviae has been demonstrated previously [36, 37, 50], but attempts to extract PCR amenable DNA from field collected mosquito exuviae were not previously successful [31]. Factors including ultraviolet B radiation (UVB), pH, salinity and presence of microorganisms can all influence degradation rate of environmental DNA (eDNA) [51, 52]. Here, average temperature and light intensity varied between the two sampling years, which could have influenced the differences in nucleic acid concentrations obtained, as well as other unknown and unquantified factors such as microbial content of the water.

## Conclusions

The present study has demonstrated for the first time that *Cx. pipiens* pupal exuviae can be utilised as a non-invasive source of trace DNA for accurate biotype differentiation for at least twenty-four hours post-eclosion. Successful PCR amplification was demonstrated from both colony and field collected exuviae through various methods of processing and is therefore accessible to a wide range of projects with different levels of available resources. This method could be of particular benefit to further studies examining behavioural differences between the forms from sympatric populations and could be utilised to establish pure colonies from field derived individuals. In addition, identification using pupal exuviae may provide a useful tool for work with other cryptic insect species.

### Electronic Supplementary Material

Below is the link to the electronic supplementary material.

Supplementary Material 1

## Data Availability

All data generated or analysed during this study are included in this published article and its additional Fles.
