# Supplementary material for: Pupal Exuviae of Culex Pipiens L. (Diptera: Culicidae) Can be Utilised as a Non-Invasive Method of Biotype Differentiation
Source: Biol Proced Online. 2024 Jun 18;26:17. doi: 10.1186/s12575-024-00246-1 (PMC11186230; doi:10.1186/s12575-024-00246-1)
Supplement: Supplementary file 1 — Supplementary Material 1 [file 12575_2024_246_MOESM1_ESM.docx]

Supplementary figures:


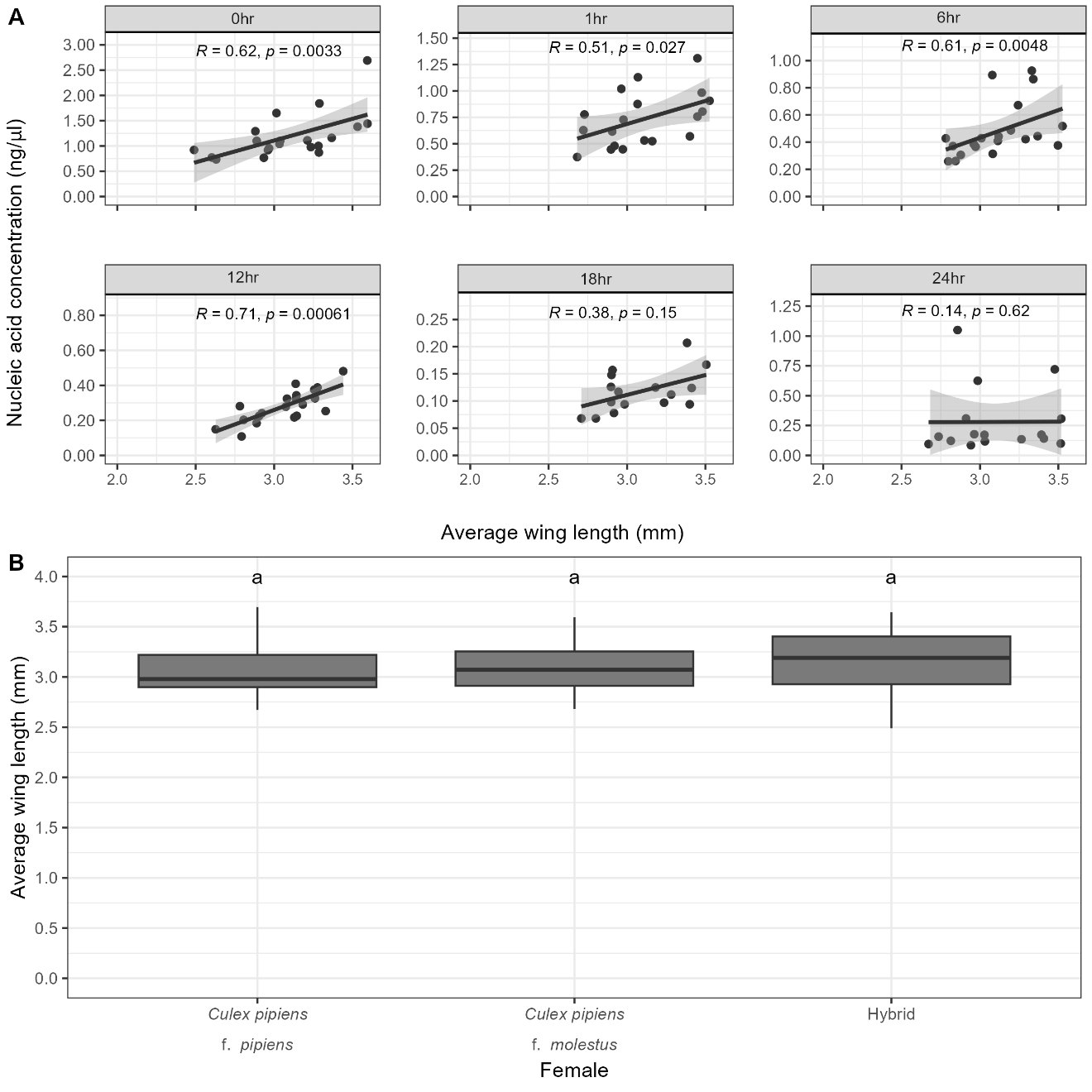


**Figure S1**. Wing length measurements of adult colony *Culex* *pipiens* used within time trial experiments A) Comparison of nucleic acid concentrations (ng/µl) with mean wing length (mm) for DNA extracts obtained from individual *Culex* *pipiens* pupal exuviae at six different time points post-eclosion. Grey shaded area indicates the 95% confidence interval. B) Comparison of wing length measurements (mm) for each of the *Culex pipiens* biotypes, according to the CQ11 assay. Horizontal black lines indicate median, 25^th^ and 75^th^ percentiles, whiskers extend to the largest and smallest values within 1.5 times the interquartile range from the 25^th^ and 75^th^ percentiles. Different letters above the boxplots indicate which groups differ significantly (*P* < 0.05) from one another.


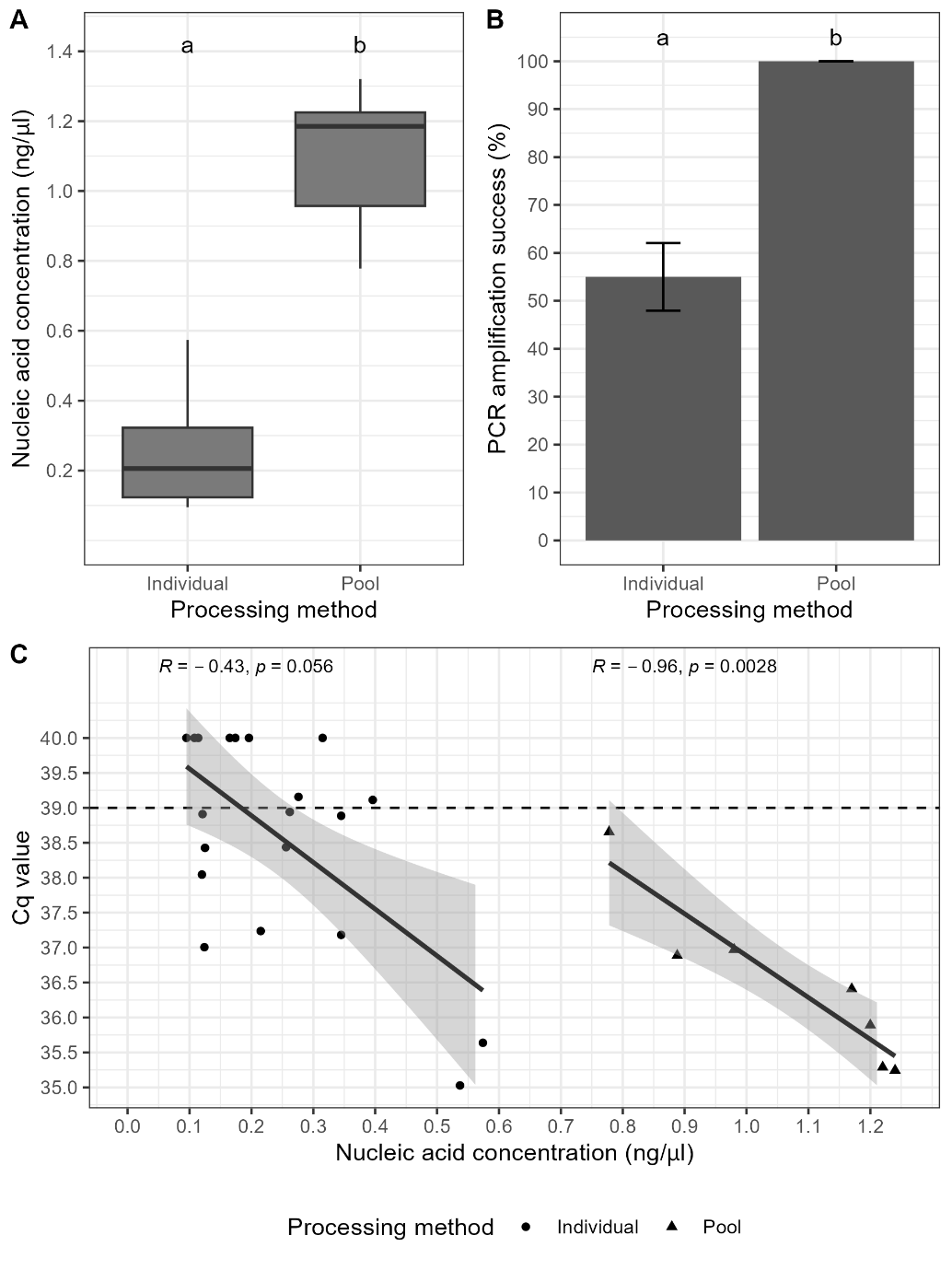


**Figure S2.** Comparison between individually and pooled processed field collected *Culex* *pipiens* pupal exuviae. A) DNA quantity (ng/µl) from DNA extracts obtained from individual and pooled field collected *Culex pipiens* pupal exuviae. Horizontal black lines indicate median, 25^th^ and 75^th^ percentiles, whiskers extend to the largest and smallest values within 1.5 times the interquartile range from the 25^th^ and 75^th^ percentiles. B) Comparison of PCR amplification success (%) for PCR extracts. Error bars indicate mean ± SD. C) Comparison of nucleic acid concentrations (ng/µl) with mean C_q_ values for PCR extracts. Grey shaded area indicates the 95% confidence interval. Different letters above the boxplots indicate which groups differ significantly (*P* < 0.05) from one another.
